# Sorption of chromium from aqueous solutions using *Fucus vesiculosus* algae biosorbent

**DOI:** 10.1186/s13065-024-01252-w

**Published:** 2024-08-07

**Authors:** Amany A. Asaad

**Affiliations:** Central Laboratory for Environmental Quality Monitoring, National Water Research Center, El-Qanater-Qalubeya, 13621 Egypt

**Keywords:** *Fucus vesiclosus*, Biosorption, Heavy metals, Chromium, Water treatment

## Abstract

The presence of heavy metals in wastewater is an environmental concern and the current treatment procedures are very expensive so it is necessary to find effective and inexpensive biosorbents. In this study, *Fucus vesiculosus* was used as a biosorbent for the biosorption of Cr(III) ions from the aqueous solutions. Biosorption parameters, such as pH, adsorbent dose, contact time, and initial concentrations of Cr(III) had the most impact on the sorption process. The required pH value for sorption was 5, the biosorbent dose was 4.0 g/L, the contact time was seen to occur after 90 min, and the Cr(III) removal decreased from 98.9 to 92%. The maximum biosorption capacity of chromium was 14.12 mg/g. FTIR analysis of *Fucus vesiculosus* biomass before the sorption process contains carboxyl, amino, hydroxyl, alkyne, and carbonyl groups, and according to the analysis after the sorption process, it was found that Cr(III) metal ions were incorporated within the sorbent during the interaction with (=C–H) active functional groups. The biosorption data were found to be perfectly suited by Langmuir equilibrium isotherm model. According to the results of this study, *Fucus vesiculosus* is an effective biosorbent for the removal of Cr(III) from aqueous solutions.

## Introduction

Heavy metals are toxic and carcinogenic, and cannot be biodegraded. Heavy metals such as zinc, copper, nickel, mercury, cadmium, lead, chromium, and arsenic have a tendency to build up in living organisms and cause a decrease in species diversity [1–3]. Metal contamination is a global environmental problem that persists and should be addressed with sufficient measures to prevent its exposure to the public. Heavy metals deposited because of industrial processes should be removed before they are received into the water since they are particularly baleful to aquatic habitats. Among these metals, chromium is one of the worthy environmental troubles that continue to cause contamination of aqueous systems and it is present in various oxidation states such as Cr(III) and Cr(VI). Chromium is used in several industries such as iron, steel, leather, metal coating, textile industry, electric power plants, coil coating, electroplating, film, photography, galvanometer, and automotive battery manufacturing industries [4–7]. The disposal of this commonly used metal in the environment causes critical pollution [8]. Moreover, searching for an important approach to remove such contaminants is an indispensable task for researchers. In this regard, various biological, physical, and chemical methods have been adopted for eliminating such heavy metals from industrial effluents such as chemical precipitation, ion exchange, and membrane separation techniques. It is preferable to use biological materials (sorbents) as an alternative method for removing chromium from aqueous solutions because the commonly used procedures for removing Cr(III) from effluents include chemical precipitation, lime coagulation, ion exchange, reverse osmosis and solvent extraction were apart from being economically expensive have disadvantages like incomplete metal removal, high reagent and energy requirements, and generation of toxic sludge or other waste products that require disposal. Efficient and environment friendly methods are thus needed to be developed to reduce heavy metal content. In this context, considerable attention has been focused in recent years upon the field of biosorption for the removal of heavy metal ions from aqueous effluents. Biosorption is a property of certain types of inactive, non-living microbial biomass to bind and concentrate heavy metals from even very dilute aqueous solution. Biomass exhibits this property, acting just as chemical substance, as an ion exchanger of biological origin [9]. Due to some benefits over conventional methods, the use of sorption materials in the removal and accumulation of heavy metals from aqueous solutions has recently received a lot of attention.

Several adsorbents have been used to remove Cr(VI) from water over the last few years, including commercial inorganic materials such as clay, silica gel, zeolite, alumina, and activated carbon, as well as bio products [10, 11] which may be alive or dead, and their effectiveness is determined by their loading capacity, selectivity, affinity, and rate of ion adsorption [12]. Lignocelluloses materials, in general, are piquing researchers’ interest due to their simple design, ease of handling, cheap operating costs, ease of availability, eco-friendliness, efficiency, and production of minimal toxic chemicals and biological sludge [13]. Furthermore, these materials are abundant in poly functional groups, which can contribute significantly to the selective adsorption of Cr(VI) from aqueous solutions [14]. Another notable property of biosorbents is their ability to convert Cr(VI) to Cr(III) at lower pH values and to totally remove Cr(VI) at moderate concentrations [15]. The removal ability of the biosorbents is affected by parameters such as pH, adsorbent dose, size, concentration, and contact time during the process. 

Various studies indicate that non-living sorbents are more effective for binding metals than biological sorbents [7]. Marine algae are known to possess excellent mineral binding capacity in different bio-selective procedures. The cell membranes of brown algae are usually composed of cellulose and algal acid, which is a straight-chain polysaccharide with a carboxyl group (–COOH) primarily responsible for binding to minerals, while sulfated polysaccharide algae bind to ion salts.. Because of these properties, algae (sorbents) are a good choice for adsorbing metal ions from the aqueous solutions in a short period and reducing heavy metal concentricity to the ppb range [16]. The sorption mechanism of heavy metals on biosorbents is thought to involve one or more of these: ion exchange, biosorption, complexation, partial precipitation formation, chelation, and electrostatic interaction. However, the most significant way that heavy metal ions are adsorbed by algae is through the ion exchange process [17]. The presence of sulfate groups, as well as a large number of carboxylic groups in brown marine algae, has been attributed to the biosorption of trivalent metal cations [18]. The characterization of the biosorbent structure and exploration of the reaction mechanism of sorbate ions and biosorbents can be identified with the help of FTIR (Fourier transform infrared) spectroscopy and SEM (A scanning electron microscope coupled with an energy dispersive spectrometer) is important to determine the structure of the *Fucus vesiculosus * [19, 20]. The reports on this kind of biosorbents and their utilization in eliminating heavy metals are few [21].

This paper investigates the biosorption capacity of *Fucus vesiculosus* (algae known by different common names such as bladder wrack, black tang, rockweed, sea grapes, sea oak, cut weed, and rock wrack) and the chromium affinity toward it. To determine the best conditions for biosorption, the effects of pH, equilibrium time, and initial concentrations were examined. FTIR spectroscopy is used to determine the specific functions involved in the association of chromium with this type of algae.

## Experimental methods

### Preparation and analysis of biomass 

Sun-dried *Fucus vesiculosus* brown algae were brought from a local market in Cairo and then dried and grounded by an electrical grinder to conduct the biosorption procedures. FTIR spectroscopy was used to determine the effective groups that chromium can occupy before and after the biosorption. The spectra ranges were 600–4000 cm^−1^ using “Thermo Fisher Nicolet 50 spectroscopy” [22]. A scanning electron microscope (SEM) is one of the common methods for imaging the microstructure and morphology of the materials [23].

### Preparation of metal solution

The preparation of 20 mg/L Cr(III) solution was carried out by diluting the working standard solution 1000 mg/L (CrCl_2_ Merck) to different concentrations from 10 to 50 mg/L. The pH values of those prepared solutions were adjusted using 1N NaOH and/or 1N HCl.

### Biosorption experiment

Biosorption of chromium by (*Fucus*) was performed by contacting 4g/L of *Fucus* with chromium concentration(20 mg/L) in a 1000 cm^3^ Pyrex conical flask intermittently for 90 min on the stirrer at 300 rpm. The mixture was filtered, and the residual concentration of the filtrate was analyzed using Inductively coupled plasma optical emission spectrometry (ICP-OES) which is well suited for such analysis because it is precise for lower concentrations [24]. The adsorbed amount of chromium (mg/g) was calculated using the following formula:

1 $${q}_{e}=\frac{\left({C}_{o}-{C}_{e}\right)V}{\text{M}},$$

where the equilibrium biosorption capacity of *Fucus* for chromium is denoted by q_e_ (mg/g), the weight of the biosorbent is symbolized by M(g), and the sorbate volume is symbolized by V(L). C_o_ and C_e_ represent the metal concentration before and after sorption (mg/L). Hence the chromium uptake ratio can be evaluated by the next equation:

2 $$R\text{\%}=\frac{\mathbf{C}\mathbf{o}-\mathbf{C}\mathbf{e}}{\mathbf{C}\mathbf{o}} \times 100.$$

## Effects of operational parameters

The determination of the optimal adsorption parameters such as pH of the biosorption solution, dose of biosorbent, biosorption time, and concentration of the adsorbate solution were essential in knowing the biosorption efficiency of the biosorbent under equilibrium condition [25]. The optimal effective adsorption parameters determined when equilibrium occurs can be achieved by preparing a series of a series of chromium solutions with pH values from 2 to 7 at a concentration of 20 mg/L, a shaking speed of 300 rpm, and a doses of (0.5, 0.1, 0.2 and 0.3) g of the adsorbent (*Fucus vesiculosus *) at 25 °C. The pH adjustment was performed using 1 N NaOH and/or 1 N HCl solutions. To establish the optimal contact time for biosorption studies, 0.2 g of *Fucus vesiculosus * powder was added to 50 mL of chromium solution at a concentration of 20 mg/L for 10–120 min at 25 °C. After the sorption process, the samples were filtered off through 0.45 μm membrane filter paper. Also the effect of biosorbent dose was investigated in the 0.05–0.3 g range. This was performed by adding a specific dose of 50 mL of chromium solution (20 mg/L) and shaking it for 90 min. After that, the sorption capacity of *Fucus vesiculosus * was calculated using the aforementioned equations. The impact of chromium initial concentration on the biosorption capacity of the biosorbent was studied by utilizing 0.2 g of Fucus powder and various concentrations of chromium solution (10, 20, 30, 40, and 50 mg/L) for 90 min at 25 °C and pH 5. 

## Biosorption isotherm models

Biosorption isotherms were employed to determine the biosorption behavior of the biosorbent and to provide a connection between the sorbate concentration C_e_ and the biosorption capacity q_e_ per mass unit of the biosorbent at equilibrium. Langmuir isotherm shows that the biosorption occurs on a homogeneous monolayer containing large biosorption sites [26]. The linear form of the Langmuir equation is presented as the following:

3 $$\frac{{C}_{e}}{{q}_{e}}=\frac{1}{{q}_{m}{K}_{L}}+\frac{{C}_{e}}{{q}_{m}},$$

where q_e_ is the adsorption capacity at equilibrium, C_e_ represents the equilibrium concentrations of chromium, q_m_ is the maximum adsorption capacity at equilibrium and K_L_ is Langmuir constant which indicates the adsorption energy. The basic characteristics of the Langmuir isotherm can be described in terms of dimensionless factor R_L_, which is assumed by:

4 $${R}_{L}=\frac{1}{(1+b{C}_{o})}$$

C_o_ is the initial concentration of adsorbate and R_L_ explains the adsorption preference of this isotherm and indicates whether the adsorption is irreversible if R_L_ = 0, linear if R_L_ = 1, or unfavorable if R_L_ > 1. 

Freundlich isotherm postulates that biosorption occurs at the available locations on heterogeneous surfaces [27]. The correlation factor R^2^ is used to evaluate the applicability of an isothermal model. The known logarithmic form of the Freundlich model is presented in Eq. (5).

5 $$\text{ln}{q}_{e}=\text{ln}{qK}_{F}+\frac{1}{\text{n}}\text{ln}{C}_{e},$$

where q_e_ and C_e_ are the capacity of biosorption (mg/g) and the concentration of sorbate (mg/L) at equilibrium, 1/n is related to the intensity of biosorption, *K*_*F*_, and n are constants.

Temkin model postulates the interactions of adsorbent-sorbate. It exhibits that the heat of an adsorbed substance is reduced linearly than logarithmically [28]. This model is characterized by a uniform binding energy distribution up to maximum binding energy and it is implemented by plotting q_e_ against lnC_e_ and then the constants can be calculated from their slope and intercept.

6 $${q}_{e}={\upbeta }_{\mathsf{T} }{\text{lnK}}_{\mathsf{T} }+{\upbeta }_{\mathsf{T} }{\text{lnC}}_{\text{e}},$$

where q_e_ denotes the quantity of adsorbed molecules that reach a state of equilibrium (mg/g); C_o_ is related to the concentration of the metal (mg/L). β constant is linked to the heat of biosorption, while R is the gas constant (8.314 J/mol K), and K represents the Temkin isotherm constant (L/g) [29].

## Biosorption kinetic models

Normally, the simulation of biosorption kinetics and evaluation of the reaction rates involve the utilization of the pseudo-first-order, pseudo-second-order, and Elovich kinetic models. The pseudo-first-order kinetic model clarifies the correlation between the adsorbent sorption sites that are occupied and the number of unoccupied sites but The relation between the adsorption capacity of the adsorbent and the time established by the pseudo-second-order kinetic model [30]. Equations (7) and (8) provide the mathematical expressions for the pseudo-first-order and pseudo-second-order, respectively [31].

7 $$log\left({q}_{e}-{q}_{t}\right)=log{q}_{e}-\left(\frac{{K}_{1}}{2.303}\right),$$

8 $$\frac{t}{{q}_{t}}=\frac{1}{{K}_{2}{{q}_{e}}^{2}}+\frac{t}{{q}_{e}},$$

where q_e_ is the amount of chromium adsorbed onto adsorbent at equilibrium in (mg/g), q_t_ is the amount chromium adsorbed onto adsorbent at any time in (mg/g), and K_1_ is the kinetics rate constant of the pseudo-first-order model (min^−1^). K_2_ is the kinetics rate constant of the pseudo-second-order model (g mg^−1^ min^−1^).

Elovich model is utilized to explain the kinetics of chemical biosorption of gas onto solid adsorbents, but it has been proven to be effective in describing various types of biosorption [32]. The following equation illustrates the Elovich model:

9 $${q}_{t}=\frac{1}{b}\text{ln}\left(ab\right)+\frac{1}{b}\text{ln}t,$$

where q_t_ (mg/g) is the adsorbate quantity at time t, a is a chemisorption rate constant and b is a constant that represents the amplitude of surface coverage and they can be calculated from the relation between their slope and intercept by plotting q_t_ versus lnt. a (mg/g min^−1^) represents the initial rate of sorption, and b (g/mg) represents the desorption constant.

## Results and discussion

### SEM analysis 

**Fig. 1:**
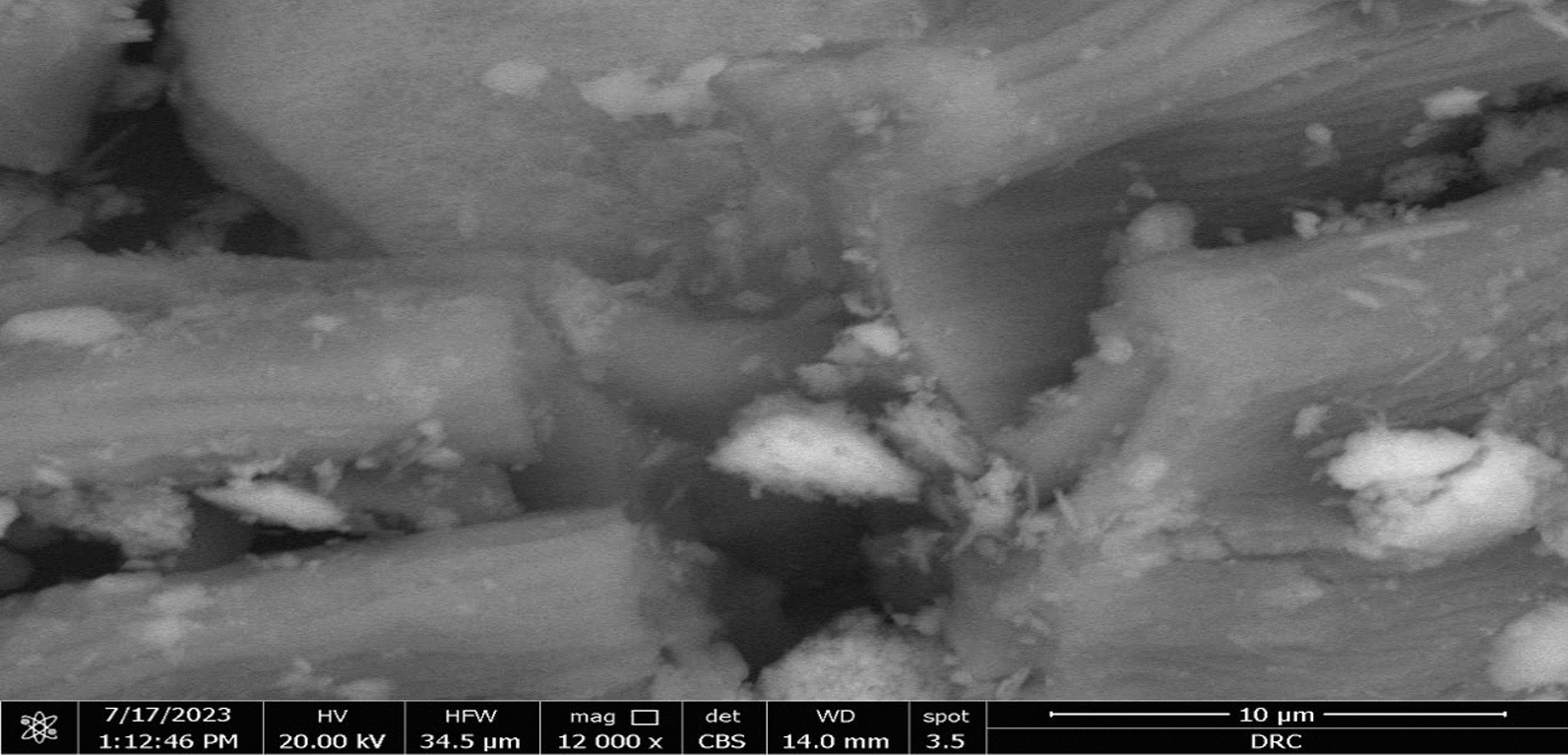
Scanning electron micrograph of dried *Fucus vesiculosus* brown algae

The surface morphology and initial formation of this species of algae have been found to have rough surfaces with pores of various sizes and shapes, increasing the surface area for metal ions to interact as shown in Fig. 1.

### FT-IR analysis

**Fig. 2:**
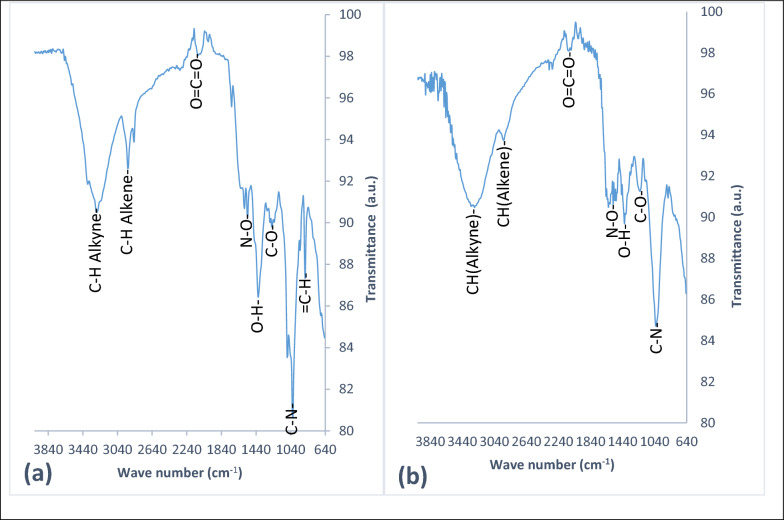
**a**, **b** FT-IR spectrum of *Fucus vesiculosus* before and after Cr(III) sorption

*Fucus vesiculosus* dried biomass before and after the sorption of chromium was analyzed using Fourier transform infrared (FTIR) spectroscopy to identify how metal ions and surface biomass interact. *Fucus vesiculosus* algae contain polysaccharides that include many negative charges and functional groups that can interact with chromium, and these functional groups include carboxylate, hydroxyl, amino, and nitro groups [33]. The spectra of adsorbents before and after chromium uptake were measured from 600 to 4000 cm^−1^ wavenumber [34]. The spectra of *Fucus vesiculosus* before the biosorption process showed different absorption bands at 3280, 2922, 2318, 1259, and1080 cm^−1^ were shifted to 3287, 2944, 1625, 1220, and 1029 cm^−1^, respectively, after biosorption of chromium. This result indicated chemical bonding among binding sites on *Fucus* biomass and the chromium [35]. The sorption bands of the sorbent at 1535 and 1416 cm^−1^ remain unchanged after the sorption process while those at 1013 and 872 cm^−1^ disappeared (Fig. 2a, b). The vibrational bands in the pure biomass of *Fucus vesiculosus* before chromium sorption at 3280 cm^−1^ and 2922 cm^−1^ are assigned to (C–H stretching) alkyne and alkene groups [36]. The vibrational band at 2318cm^−1^ is related to carbon dioxide (O=C=O). The band at 1535 cm^−1^ is due to N–O functional group [37]. The sharp band at 1416 cm^−1^ is probably due to the bending vibration of the hydroxyl group (O–H) [38]. The vibrational band at 1259 cm^−1^ is restricted to (C–O) stretching [39]. The band at 1080 cm^−1^ relates to the (C–N) stretching mode [40]. The bands between the wavenumbers of 1800–750 cm^−1^ (fingerprint regions) reflected the biochemical compositions, especially the moieties of carbohydrate, lipid, protein secondary and polyphenols [41] The band at 860cm^−1^ is due to =C–H bending disappeared in the FTIR spectrum for the biomass sample of *Fucus vesiculosus* after the chromium sorption process [42]. Figure 2b demonstrated that the chromium was incorporated within the sorbent during the interaction with the active functional groups (=C–H) [43].

### Biosorption studies

**Fig. 3:**
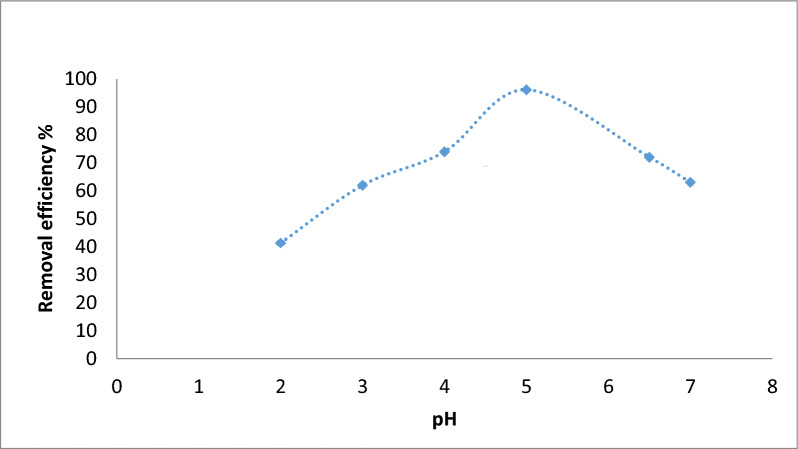
Effect of pH on Cr(III) sorption

**Fig. 4:**
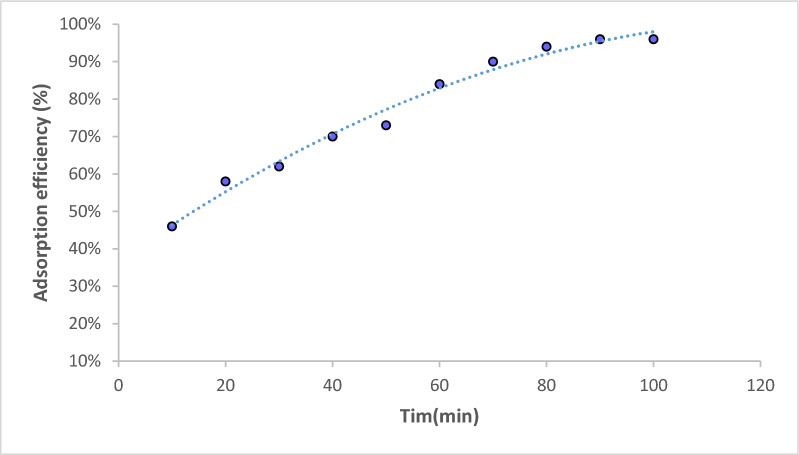
Effect of contact time on Cr(III) sorption

**Fig. 5:**
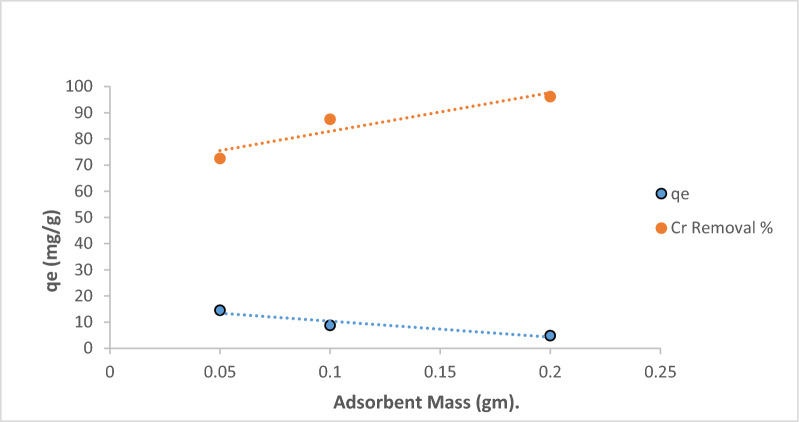
Effect of biosorbent mass on Cr(III) sorption

**Fig. 6:**
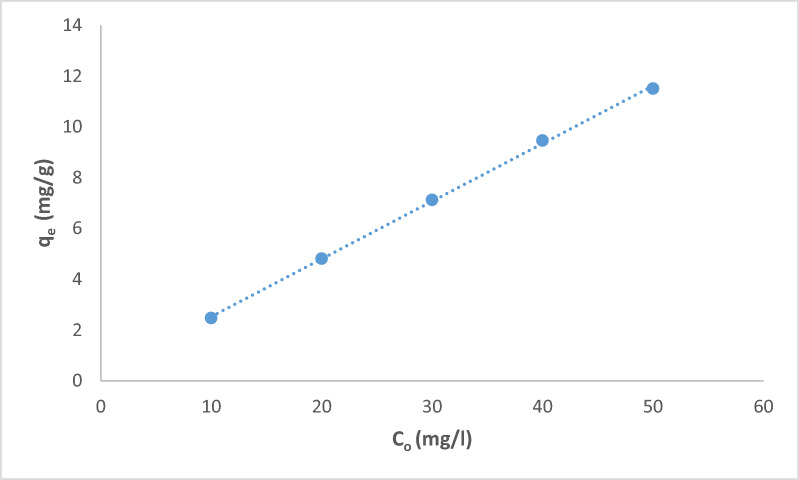
Effect of initial concentration Cr(III) sorption

The pH of the contact solution is an important parameter controlling the biosorption process. The variation of pH values changes the solution acidity or basicity and affects the *Fucus* surface charge. The pH of the initial chromium concentration (20 mg/L) varied from 2.0 to 9.0 at a constant dose (4 g/L) and the stirring rate at 300 rpm for 90 min at room temperature (25 °C), as shown in Fig. 3. The sorption of chromium was raised by increasing pH ranges from 2.0 to 5.0 where the capacity removal percentage of chromium reached 96.15%. It may be due to the active functional groups in the sorbent that facilitate biosorption by participating in metal ion binding [44]. This was followed by a gradual decrease in the chromium removal % at pH values greater than 5.0. The sorbent mass was then varied (0.05–0.3 g/50mL) for an initial Cr(III) concentration of 20 mg/L at 25 °C and pH 5.0 for 90 min as shown in Fig. 4. The sorption of Cr(III) increased with an increase in sorbent mass at an equilibrium time of 90 min from 46 to 96% and the equilibrium biosorption capacity reached 29 mg/g. This is because the higher the initial concentration of the metal ions, the higher the chance of collisions with adsorption sites on the surface of the adsorbent. Moreover, the driving force of mass transfer is better, which is conducive to reduce the mass transfer resistance and increase the biosorption capacity [45]. Figure 5 shows the removal of Cr(III) which was accomplished within 90 min so there is no any additional sorption and an equilibrium state is reached. The rate of the biosorption process will increase significantly with increasing contact time so that it reaches the equilibrium point. Where, the longer the contact time, the greater the adsorption capacity. Figure 6 explains the effect of Cr(III) concentrations on the sorption process under study in which the higher uptake occurred at 90 min under equilibrium [46]. It may be imputed to the consumption of the available sites of the sorbent stable amount at equilibrium. Therefore, 90 min is the time required time for the sorption process. The sorption of Cr(III) declined from 10 mg/L to 50 mg/L and there was a significant rising in q_e_ of Cr(III) at the *Fucus* surface when the Cr(III) concentration ascents from 2.5 to 11.5 mg respectively. Hence the decrease in percentage removal from 98.9% to 92% may be attributed to the lack of other available sites of the sorbent and there is a repulsion force between the sorbate and bulk phase which reduces the uptake of the chromium [47, 48].

#### Biosorption isotherms

**Fig. 7:**
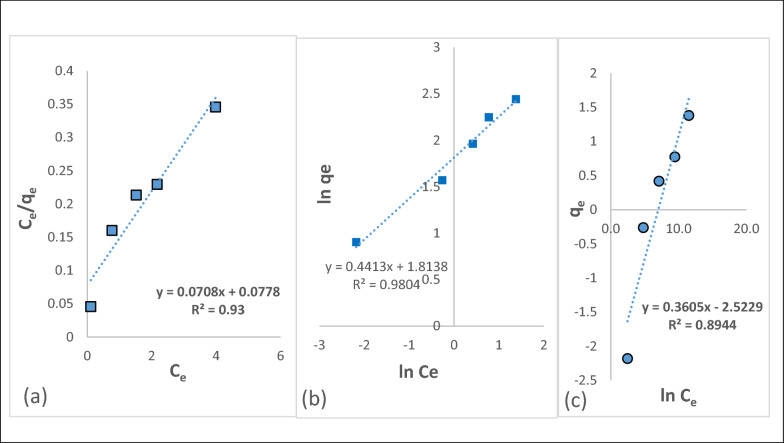
**a**–**c** Langmuir, Freundlich and Temkin Isotherms for the sorption of Cr(III) on the *Fucus vesiculosus*

The biosorption isotherms are commonly used to reflect the performance of biosorbents in biosorption processes. Langmuir isotherm is useful for monolayer adsorption, the Freundlich isotherm shows adsorption on the heterogeneous surfaces of adsorbate-adsorbent systems and the Temkin isotherm model assumes that the adsorption energy of all molecules decreases linearly with increasing adsorbent surface occupancy. In this research, the biosorption isotherms were achieved for chromium solutions of different initial concentrations from 10 to 50 mg/L, an algae dose of 4 g/L at 300 rpm for 90 min, and at pH 5 [49–51]. The concentration of adsorbed chromium was determined according to Eq. (3). Figure 7a–c represented the biosorption isotherms of Cr(III) by the *Fucus* surface at pH 5 using the Langmuir, Freundlich, and Temkin models, respectively. Isotherm parameters are reported in Table 1. The Langmuir constant values (K_L_) show that strong interactions between metal ions and apparent functional groups are involved in the biosorption processes, regardless of the nature of metal ions or biosorbent. The separation factor in Langmuir isotherm (R_L_) was less than one and the correlation factor (R^2^) was 99%, which showed that the biosorption process was favorable [52]. The parameter 1/n in the Freundlich model is less than unity indicating that all biosorption processes are favourable. Moreover, the obtained values have better performance in biosorption of chromium(III) metal ions. These observations are also supported by the Temkin model parameters (Table 1), which show that in the biosorption process, the retention of metal ions is achieved through strong interactions, confirming the removal efficiency trend.

**Table 1:** Fitting parameters of isotherm models for the biosorption of Cr(III) on the *Fucus vesiculosus* biosorbent

Models	Parameters	Values
	q_max_	17.06 mg/g
	K_L_	0.52 L/mg
Langmuir	R_L_	0.16
	R^2^	0.99
	K_f_	5.39 mg/g
Freundlich	n	1.7
	R^2^	0.98
	β_T_	0.27
Temkin	K_T_	1.97 L/g
	R^2^	0.97

#### Biosorption kinetics

**Fig. 8:**
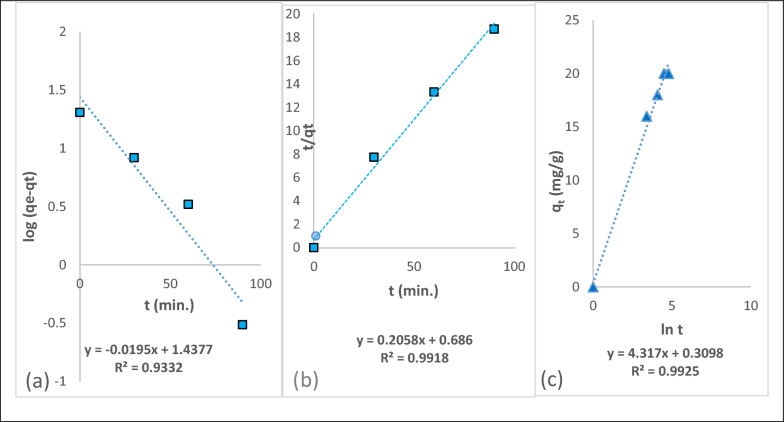
**a**–**c** Pseudo first order, pseudo second order and Elovich kinetic models for the sorption of Cr(III) on the *Fucus vesiculosus* biosorbent

The kinetics of the Cr(III) biosorption process was evaluated using different kinetic models. Pseudo-first, second-order kinetics, and Elovich models were applied as shown in (Fig. 8a–c) and the estimated kinetic parameters have been illustrated in Table 2. The appropriate kinetic model of Cr(III) biosorption was governed by the linear correlation coefficient (R^2^) values taken from model plots. The value of R^2^ in the pseudo-second-order kinetic model (0.991) was higher than the value of R^2^ in the pseudo-first-order (0.933), hence it may be attributed to chemically induced biosorption kinetics including valence strength via ion exchange or through the electron interactions between adsorbed molecules on the *Fucus* surface and the adsorbent [53, 54]. It has been achieved that the high correlation coefficient indicate a good fit of experimental data to the pseudo-second-order model [55]. Also, this indicated that the rate constant (K_2_) of Cr(III) was 0.062 g/mg min, which reveals that the pseudo-second-order kinetic model is based on the assumption that the rate-limiting step is chemical sorption or chemisorption and predicts the behavior over the whole range of adsorption. In this condition, the adsorption rate is dependent on adsorption capacity not on concentration of adsorbate [56]. Moreover, in order to comprehend the characteristics of chemisorption, the Elovich model has been utilized. The amounts of (1/b) and (1/b) ln (ab) have been evaluated by the slope and intercept of the linear correlation [57, 58]. The value of (1/b) represents the number of available sites required for biosorption, while the biosorption quantity is indicated by the value of (1/b) ln (ab). Elovich model data has been demonstrated in (Table 2).

**Table 2:** Kinetic parameters for the biosorption of Cr(III) on the *Fucus vesiculosus* biosorbent

Models	Parameters	Values
	q_e_	20.30 mg/g
Pseudo-Frist-Order	K_1_	0.0449 min^−1^
	R^2^	0.93
	q_e_	23.62 mg/g
Pseudo-Second-Order	K_2_	0.062 g/mg. min
	R^2^	0.99
	q_t_	14.80 mg/g
Elovich	a	4.63 mg/g min^−1^
	b	0.23 g/mg
	R^2^	0.99

## Conclusions

This study presents a new approach using *Fucus vesiculosus* algae to remove chromium from aqueous solutions in a safe and environmental manner. The maximum chromium removal capacity was 96.15% at pH 5, and dose (4.0 g/L) in 90 min. The biosorption models described the biosorption equilibrium of chromium with *Fucus vesiculosus*, the maximum biosorption capacity of chromium was 14.12 mg/g and the isothermal constants were determined. The obtained results confirmed that the biosorption equilibrium data are excellently integrated into the Langmuir model and also the pseudo-second-order equation gave an excellent correlation between the experimental and the calculated data in the biosorption of Cr(III). Finally, it was concluded that *Fucus vesiculosus* is an effective and environmentally friendly biosorbent and a suitable candidate for the removal of Cr(III) from aqueous solutions. 

## Data Availability

The datasets generated during and/or analyzed during the current study are available from the corresponding author on reasonable request.

## References

[1] Duffus JH. “Heavy metals”—a meaningless term? (IUPAC technical report). Pure Appl Chem. 2002;74(5):793–807. 10.1351/pac200274050793. 10.1351/pac200274050793

[2] Onakpa MM, Njan AA, Kalu OC. A review of heavy metal contamination of food crops in Nigeria. Ann Glob Heal. 2018;84(3):488–94. 10.29024/aogh.2314. 10.29024/aogh.2314 PMC6748284 30835390

[3] Briffa J, Sinagra E, Blundell R. Heliyon Heavy metal pollution in the environment and their toxicological effects on humans. Heliyon. 2020;6(June): e04691. 10.1016/j.heliyon.2020.e04691. 32964150 10.1016/j.heliyon.2020.e04691 PMC7490536

[4] No CAS, No EC, Sens S, Acute A, Chronic A. Phase-out of chromium (III) in leather tanning. (Iii):1–5.

[5] Picu A, Enescu MC, Stoian EV, Petre IC. Studies and perspectives on the types of corrosion occurring in continuous chromium plating plants. Sci Bull Valahia Univ Mater Mech. 2023;19(21):49–57. 10.2478/bsmm-2023-0019. 10.2478/bsmm-2023-0019

[6] Wint N, Warren DJ, DeVooys ACA, McMurray HN. The use of chromium and chromium (III) oxide PVD coatings to resist the corrosion driven coating delamination of organically coated packaging steel. J Electrochem Soc. 2020;167(14): 141506. 10.1149/1945-7111/abc360. 10.1149/1945-7111/abc360

[7] Sharma S, Malaviya P. Bioremediation of tannery wastewater by chromium resistant fungal isolate fusarium chlamydosporium SPFS2-g. Curr World Environ. 2014;9(3):721–7. 10.12944/CWE.9.3.21. 10.12944/CWE.9.3.21

[8] Yalçin S, Apak R, Hizal J, Afşar H. Recovery of copper (II) and chromium (III, VI) from electroplating-industry wastewater by ion exchange. Sep Sci Technol. 2001;36(10):2181–96. 10.1081/SS-100105912. 10.1081/SS-100105912

[9] Vinodhini V, Das N. Biowaste materials as sorbents to remove chromium (VI) from aqueous environment—a comparative study. ARPN J Agric Biol Sci. 2009;4(6):19–23.

[10] Basnet P, Gyawali D, Nath Ghimire K, Paudyal H. An assessment of the lignocellulose-based biosorbents in removing Cr(VI) from contaminated water: a critical review. Results Chem. 2022;4(June): 100406. 10.1016/j.rechem.2022.100406. 10.1016/j.rechem.2022.100406

[11] Sankaran S, Khanal SK, Jasti N, Jin B, Pometto AL, Van Leeuwen JH. Use of filamentous fungi for wastewater treatment and production of high value fungal byproducts: a review. Crit Rev Environ Sci Technol. 2010;40(5):400–49. 10.1080/10643380802278943. 10.1080/10643380802278943

[12] Pertile E, Dvorský T, Václavík V, Heviánková S. Use of different types of biosorbents to remove Cr(VI) from aqueous solution. Life. 2021;11(3):240. 10.3390/life11030240. 33799430 10.3390/life11030240 PMC8000416

[13] Michalak I, Godlewska K, Marycz K. Biomass enriched with minerals via biosorption process as a potential ingredient of horse feed. Waste Biomass Valorization. 2019;10(11):3403–18. 10.1007/s12649-018-0351-5. 10.1007/s12649-018-0351-5

[14] Portal G. Preparation and properties of principal TL products. Appl Thermolumin Dosim. 1981;852745443:97–122.

[15] Villabona-Ortíz A, Tejada-Tovar C, González-Delgado ÁD. Elimination of chromium (VI) and nickel (II) ions in a packed column using oil palm bagasse and yam peels. Water (Switzerland). 2022;14(8):1240. 10.3390/w14081240. 10.3390/w14081240

[16] Reduction of heavy metal pollution using brown algae: a review. 4(4):1–6.

[17] Gouda SA, Taha A. Biosorption of heavy metals as a new alternative method for wastewater treatment: a review. Egypt J Aqua Biol Fish. 2023;27(2):135–53. 10.21608/ejabf.2023.291671

[18] Baby R, Hussein MZ, Abdullah AH, Zainal Z. Nanomaterials for the treatment of heavy metal contaminated water. Polymers (Basel). 2022;14(3):1–17. 10.3390/polym14030583. 10.3390/polym14030583 PMC8838446 35160572

[19] Sabah NH. Fourier transform. Circuit Anal with PSpice. Published online 2018:687–709. 10.1201/9781315402222-23.

[20] Zhou W, Apkarian R, Wang ZL, Joy D. Fundamentals of scanning electron microscopy (SEM). In: Scanning microscopy for nanotechnology. New York: Springer; 2007. p. 1–40. 10.1007/978-0-387-39620-0_1.

[21] Vieira RHSF, Volesky B. Biosorption: a solution to pollution? Int Microbiol. 2000;3(1):17–24. 10963329

[22] Asencios YJO, Parreira LM, Perpetuo EA, Rotta AL. Characterization of seaweeds collected from Baixada Santista litoral, and their potential uses as biosorbents of heavy metal cations. Rev Mex Ing Quim. 2022;21(1):1–22. 10.24275/rmiq/IA2600. 10.24275/rmiq/IA2600

[23] Ali A, Zhang N, Santos RM. Mineral characterization using scanning electron microscopy (SEM): a review of the fundamentals, advancements, and research directions. Appl Sci. 2023;13(23):12600. 10.3390/app132312600. 10.3390/app132312600

[24] Frois SR, Tadeu Grassi M, De Campos MS, Abate G. Determination of Cr(VI) in water samples by ICP-OES after separation of Cr(III) by montmorillonite. Anal Methods. 2012;4(12):4389–94. 10.1039/c2ay26125a. 10.1039/c2ay26125a

[25] El-Naggar NEA, Hamouda RA, Mousa IE, Abdel-Hamid MS, Rabei NH. Biosorption optimization, characterization, immobilization and application of Gelidium amansii biomass for complete Pb^2+^ removal from aqueous solutions. Sci Rep. 2018;8(1):1–19. 10.1038/s41598-018-31660-7. 30194341 10.1038/s41598-018-31660-7 PMC6128825

[26] Szostak K, Hodacka G, Długosz O, Pulit-Prociak J, Banach M. Sorption of mercury in batch and fixed-bed column system on hydrochar obtained from apple pomace. Processes. 2022;10(10):2114. 10.3390/pr10102114. 10.3390/pr10102114

[27] Ali K, Javaid MU, Ali Z, Zaghum MJ. Review article biomass-derived adsorbents for dye and heavy metal removal from wastewater. 2021;2021.

[28] Abid H, Amanat A, Ahmed D, Qamar T. Adsorption efficacy of Carissa opaca roots residual biomass for the removal of copper from contaminated water. Chem Int. 2023;9(1):1–7.

[29] Yazdani Shargh A, Hossein Sayadi M, Heidari A, Shargh YA, Green Biosynthesis HA. Green biosynthesis of palladium oxide nanoparticles using *Dictyota indica* seaweed and its application for adsorption of palladium oxide nanoparticles using *Dictyota indica* Seaweed and its application for adsorption. J Water Environ Nanotechnol. 2018;3(4):337–47. 10.22090/jwent.2018.04.006. 10.22090/jwent.2018.04.006

[30] Khalaf SM, Al-Mahmoud SM. Adsorption of tetracycline antibiotic from aqueous solutions using natural Iraqi bentonite. Egypt J Chem. 2021;64(10):5511–9. 10.21608/EJCHEM.2021.76358.3734. 10.21608/EJCHEM.2021.76358.3734

[31] Abdel Ghafar HH, Ali GAM, Fouad OA, Makhlouf SA. Enhancement of adsorption efficiency of methylene blue on Co_3_O_4_/SiO_2_ nanocomposite. Desalin Water Treat. 2015;53(11):2980–9. 10.1080/19443994.2013.871343. 10.1080/19443994.2013.871343

[32] Ruíz-Baltazar de ÁJ, Reyes-López SY, Mondragón-Sánchez de ML, Robles-Cortés AI, Pérez R. Eco-friendly synthesis of Fe_3_O_4_ nanoparticles: evaluation of their catalytic activity in methylene blue degradation by kinetic adsorption models. Results Phys. 2019;12(1):989–95. 10.1016/j.rinp.2018.12.037. 10.1016/j.rinp.2018.12.037

[33] El-Naggar NEA, El-khateeb AY, Ghoniem AA, El-Hersh MS, Saber WEIA. Innovative low-cost biosorption process of Cr^6+^ by Pseudomonas alcaliphila NEWG-2. Sci Rep. 2020;10(1):1–18. 10.1038/s41598-020-70473-5. 32820181 10.1038/s41598-020-70473-5 PMC7441394

[34] Kayranli B, Gok O, Yilmaz T, et al. Low-cost organic adsorbent usage for removing Ni^2+^ and Pb^2+^ from aqueous solution and adsorption mechanisms. Int J Environ Sci Technol. 2022;19(5):3547–64. 10.1007/s13762-021-03653-z. 10.1007/s13762-021-03653-z

[35] Sartape AS, Mandhare AM, Jadhav VV, Raut PD, Anuse MA, Kolekar SS. Removal of malachite green dye from aqueous solution with adsorption technique using *Limonia acidissima* (wood apple) shell as low cost adsorbent. Arab J Chem. 2017;10:S3229–38. 10.1016/j.arabjc.2013.12.019. 10.1016/j.arabjc.2013.12.019

[36] Adeniyi AG, Ighalo JO, Onifade DV. Biochar from the thermochemical conversion of orange (*Citrus sinensis*) peel and albedo: product quality and potential applications. Chem Africa. 2020;3(2):439–48. 10.1007/s42250-020-00119-6. 10.1007/s42250-020-00119-6

[37] Castro L, Bonilla LA, González F, Ballester A, Blázquez ML, Muñoz JA. Continuous metal biosorption applied to industrial effluents: a comparative study using an agricultural by-product and a marine alga. Environ Earth Sci. 2017;76(14). 10.1007/s12665-017-6803-6.

[38] Hossain UH, Seidl T, Ensinger W. Combined in situ infrared and mass spectrometric analysis of high-energy heavy ion induced degradation of polyvinyl polymers. Polym Chem. 2014;5(3):1001–12. 10.1039/c3py01062g. 10.1039/c3py01062g

[39] Hsieh W-H, Cheng WT, Chen L-C, Lin HL, Lin SY. Biomedical and pharmaceutical sciences non-isothermal dehydration kinetics of glucose monohydrate, maltose monohydrate and trehalose dihydrate by thermal analysis and DSC-FTIR study. Mater Sci Chem. 2018;1(1):1–6.

[40] Yang L, May PW, Yin L, Smith JA, Rosser KN. Ultra fine carbon nitride nanocrystals synthesized by laser ablation in liquid solution. J Nanoparticle Res. 2007;9(6):1181–5. 10.1007/s11051-006-9192-4. 10.1007/s11051-006-9192-4

[41] Lu X, Wang J, Al-Qadiri HM, et al. Determination of total phenolic content and antioxidant capacity of onion (*Allium cepa*) and shallot (*Allium oschaninii*) using infrared spectroscopy. Food Chem. 2011;129(2):637–44. 10.1016/j.foodchem.2011.04.105. 30634280 10.1016/j.foodchem.2011.04.105

[42] Porto ICCM, Nascimento TG, Oliveira JMS, Freitas PH, Haimeur A, França R. Use of polyphenols as a strategy to prevent bond degradation in the dentin–resin interface. Eur J Oral Sci. 2018;126(2):146–58. 10.1111/eos.12403. 29380895 10.1111/eos.12403

[43] Tytłak A, Oleszczuk P, Dobrowolski R. Sorption and desorption of Cr(VI) ions from water by biochars in different environmental conditions. Environ Sci Pollut Res. 2015;22(8):5985–94. 10.1007/s11356-014-3752-4. 10.1007/s11356-014-3752-4 PMC4381096 25378029

[44] Yang X. Surface functional groups of carbon-based adsorbents and their roles in the removal of heavy metals from aqueous solutions: a critical review. Chem Eng J. 2019;352:1–69. 10.1016/j.cej.2019.02.119 PMC8437042 34522159

[45] Jiang J, Trundle P, Ren J, et al. We are IntechOpen, the world ’ s leading publisher of Open Access books Built by scientists, for scientists TOP 1 %. INTECH. 2010;34(8):57–67.

[46] Garg R, Garg R, Sillanpää M, et al. Rapid adsorptive removal of chromium from wastewater using walnut-derived biosorbents. Sci Rep. 2023;13(1):1–12. 10.1038/s41598-023-33843-3. 37100812 10.1038/s41598-023-33843-3 PMC10133242

[47] Aworanti OA, Agarry SE. Kinetics, isothermal and thermodynamic modelling studies of hexavalent chromium ions adsorption from simulated wastewater onto Parkia biglobosa-sawdust derived acid-steam activated carbon. Appl J Envir Eng Sci. 2017;3:58–76.

[48] Liu Y, Li X, Wang Y, Zhou J, He W. Preparation and characterization of *Camellia oleifera* nut shell-based bioadsorbent and its application for heavy metals removal. BioResources. 2019;14(1):234–50. 10.15376/biores.14.1.234-250. 10.15376/biores.14.1.234-250

[49] Stefanne C, Costa D, Galdeano B, et al. Equilibrium study of binary mixture biosorption of Cr (III) and Zn (II) by dealginated seaweed waste : investigation of adsorption mechanisms using X-ray photoelectron spectroscopy analysis. Environ Sci Pollut Res. 2019;26:28470–80. 10.1007/s11356-018-2880-7 30091076

[50] Manzoor Q, Sajid A, Hussain T, Iqbal M, Abbas M, Nisar J. Efficiency of immobilized Zea mays biomass for the adsorption of chromium from simulated media and tannery wastewater. J Mater Res Technol. 2019;8(1):75–86. 10.1016/j.jmrt.2017.05.016. 10.1016/j.jmrt.2017.05.016

[51] Olafadehan OA, Akpo OY, Enemuo O, Amoo KO, Abatan OG. Equilibrium, kinetic and thermodynamic studies of biosorption of zinc ions from industrial wastewater using derived composite biosorbents from walnut shell. African J Environ Sci Technol. 2018;12(9):335–56. 10.5897/ajest2018.2515. 10.5897/ajest2018.2515

[52] Wang Y, Dou P, You X, et al. Naphthenic acids removal from model transformer oil by diethylamine modified resins. Molecules. 2023;28:2444. 36985416 10.3390/molecules28062444 PMC10054115

[53] Orozco CI, Freire MS, Gómez-díaz D, González-álvarez J. Removal of copper from aqueous solutions by biosorption onto pine sawdust. Sustain Chem Pharm. 2022;2023(32): 101016. 10.1016/j.scp.2023.101016. 10.1016/j.scp.2023.101016

[54] Arshadi M, Amiri MJ, Mousavi S. Kinetic, equilibrium and thermodynamic investigations of Ni(II), Cd(II), Cu(II) and Co(II) adsorption on barley straw ash. Water Resour Ind. 2014;6:1–17. 10.1016/j.wri.2014.06.001. 10.1016/j.wri.2014.06.001

[55] Wang H, Wang W, Zhou S, Gao X. Adsorption mechanism of Cr(VI) on woody-activated carbons. Heliyon. 2023;9(2): e13267. 10.1016/j.heliyon.2023.e13267. 36798761 10.1016/j.heliyon.2023.e13267 PMC9925964

[56] El Refay HM, Goma A, Badawy N, Al Zahra GF. Agricultural by-products as green chemistry in elimination of reactive red 43 from aqueous media-adsorption properties and thermodynamics study. Egypt J Chem. 2022;65(8):103–14. 10.21608/ejchem.2022.102614.4757. 10.21608/ejchem.2022.102614.4757

[57] Tattibayeva Z, Tazhibayeva S, Kujawski W, Zayadan B, Musabekov K. Peculiarities of adsorption of Cr (VI) ions on the surface of Chlorella vulgaris ZBS1 algae cells. Heliyon. 2022;8(9): e10468. 10.1016/j.heliyon.2022.e10468. 36105478 10.1016/j.heliyon.2022.e10468 PMC9465124

[58] Krika F, Azzouz MCN. Adsorptive removal of cadmium from aqueous media using Posidonia oceanica biomass: equilibrium, dynamic and thermodynamic studies. Int J Environ Sci Technol. 2015;12:983–94. 10.1007/s13762-013-0483-x. 10.1007/s13762-013-0483-x

